# Human Skin Fibroblasts as an In Vitro Model Illustrating Changes in Collagen Levels and Skin Cell Migration Under the Influence of Selected Plant Hormones

**DOI:** 10.3390/bioengineering11121188

**Published:** 2024-11-25

**Authors:** Agata Jabłońska-Trypuć, Walentyn Pankiewicz, Elżbieta Wołejko, Gabriela Sokołowska, Jorge Estévez, Miguel A. Sogorb, Urszula Wydro

**Affiliations:** 1 Department of Chemistry, Biology and Biotechnology, Faculty of Civil Engineering and Environmental Sciences, Bialystok University of Technology, Wiejska 45E Street, 15-351 Białystok, Poland; e.wolejko@pb.edu.pl (E.W.); gabriela.sokolowska@sd.pb.edu.pl (G.S.); u.wydro@pb.edu.pl (U.W.); 2 Academy of Medical Sciences in Białystok, Krakowska Street 9, 15-875 Białystok, Poland; walentyn.pankiewicz@gmail.com; 3 Unidad de Toxicología y Seguridad Química, Instituto de Bioingeniería, Universidad Miguel Hernández de Elche, 03202 Elche, Spain; jorge.estevez@umh.es (J.E.); msogorb@umh.es (M.A.S.)

**Keywords:** collagen, fibroblasts, skin kinetin, N-6-benzyladenine, cytokinins

## Abstract

Human skin fibroblasts are an excellent in vitro model for tracking the processes occurring in human skin and studying the potential impact of various biologically active substances on these processes. Two plant hormones, which are included in the cytokinins group—kinetin (K) and N-6-benzyladenine (BA)—have a positive effect on human skin. Therefore, an attempt was made to examine the effect they have on key skin functions, cell proliferation, and migration, as well as collagen synthesis in them. The effect of phytohormones was studied at selected concentrations for kinetin—10 μM and 1 μM—and for N-6-benzyladenine—1 μM and 0.1 μM. A wound-healing assay was used in order to analyze cell migration and proliferation. The content of total protein and collagen in cells and culture medium was determined. The obtained results confirm that the studied compounds induce cell migration and proliferation, as well as collagen biosynthesis. The positive effect of kinetin and N-6-benzyladenine on fibroblast metabolism that we have demonstrated allows us to indicate them as compounds with potentially therapeutic properties. Therefore, we conclude that they should be subjected to further molecular and in vivo studies focusing on pathologies connected with skin diseases and aging.

## 1. Introduction

In vitro culture of human skin fibroblasts is an excellent research model allowing the analysis of the activity of naturally originating substances on selected parameters and morphological pathologies occurring in the skin [1,2]. Collagen is the basic protein component of the skin [3]. Considering the fact that fibroblasts are responsible for collagen synthesis, different changes in their activity are not without significance for the proper functioning of connective tissue, in which collagen is the main structural protein [4,5]. Many active substances used in therapeutic formulas and supplements have a beneficial effect on metabolic processes occurring in skin cells. Examples of such substances are selected hormones derived from plants, such as kinetin (K) and N-6-benzyladenine (BA), and K is a natural while BA is a synthetic plant hormone.

Cytokinins are one of the main and most important groups of hormones derived from plants. The molecular mechanism of their action in plant tissues has been described in significant detail, but there is still not enough literature data documenting their effect on human cell metabolism [6,7]. Our previous studies have shown a positive effect of selected compounds from the cytokinin group—kinetin (K) and N-6-benzyladenine (BA)—on the level of oxidative stress [8]. It should be emphasized that we know that the compounds tested are characterized by a positive effect on human skin fibroblasts, but it seems important that they also affect key skin proteins such as collagen. The study conducted on a healthy cell line can be an introduction to further analyses conducted on pathological cancer cell lines. In order to implement the application of kinetin and benzyladenine as future therapeutic agents against diseases associated not only with oxidative stress, but also as modulating agents associated with the proper functioning of connective tissue, it is necessary, in addition to molecular in vitro studies, to directly assess the toxicity of kinetin in vivo in an animal model. The cytokinin group also includes compounds derived from fatty acids. An example of such a compound is traumatic acid, a derivative of traumatin which is a wound hormone that stimulates wound healing in plants. Our latest studies have also confirmed the positive effect of this compound on collagen biosynthesis and the reduction in oxidative stress levels in human fibroblasts [9]. Taking into account the obtained results regarding both adenine derivatives as well as fatty acid-derived cytokinins, we wanted to study the activity of K and BA on cell migration and the synthesis of collagen, which is the basic structural protein of human skin. Since the selected cytokinins have not yet been tested for their potential effect on human skin, the aim of our research was, among other things, to assess their effect on morphological changes in cells, their migration and selected biochemical parameters, such as total protein and collagen content. By conducting these studies, we wanted to initially demonstrate whether the tested phytohormones should be studied in more detail in the context of their potential effect on human skin. We conducted research on fibroblasts, which are a healthy cell line used as a specific in vitro model allowing us to verify our hypothesis. Our hypothesis assumes that the tested compounds from the cytokinin group will improve the rate of cell proliferation, increase their migration potential, and stimulate the synthesis of key skin proteins, an example of which is collagen. It should be emphasized that these are in vitro studies constituting a kind of introduction to further molecular analyses and possible in vivo studies. They provide a basis for further considerations and detailed analyses without indicating specific application or therapeutic doses.

This work is based on studies conducted under normal physiological conditions, without the use of stress factors, using the most appropriate in vitro model—fibroblasts—which constitute the vast majority of cells that build human skin. We analyzed the compounds tested at concentrations determined in previous experiments [8]. In the previously established concentration range for each of the analyzed phytohormones, we studied their effect on the content of total protein, collagen, and on cell migration measured by the wound-healing assay method. Our goal will be to demonstrate an increase in cell proliferation and protein synthesis and stimulation of cell migration. Such a positive effect of our research will indicate the need to continue them and expand their scope to include molecular analyses and in vivo studies. The metabolic activation of fibroblasts is crucial in many situations, not only related to aging but also to many connective tissue diseases and the scarring process. Therefore, it seems important to study selected cytokinins as factors activating the synthesis of structural proteins in fibroblasts.

## 2. Materials and Methods

### 2.1. Chemical Compounds

All the reagents including kinetin, N-6-benzyladenine, PBS (phosphate-buffer saline), DMEM, FBS, Sirius Red dye (Direct Red 80), and Collagen Standard Solution (PureCol^™^-S) were obtained from Sigma Aldrich (St. Louis, MO, USA). Other reagents and solvents used in this study were of analytical grade. The plastic used for cell culture was obtained from Sarstedt (Nümbrecht, Germany). Milli-Q^®^ ultrapure water was used in all experiments. All substances added to the culture medium (K, BA, collagen and other reagents) were filtered through a 0.22 μm filter to avoid microbiological contamination. The tested compounds were dissolved in DMSO and then in culture medium so that the final DMSO concentration did not exceed 0.1% in the well.

### 2.2. Cell Line

The effect of K and BA on selected parameters was studied using normal human skin fibroblasts (CRL1474) obtained from American Type Culture Collection (ATCC), under physiological conditions, without the use of stress factors. The fibroblast cell line was cultured in DMEM medium supplemented with 10% FBS at 37 °C and 5% CO_2_. Fibroblasts seeded at a density of 1 × 10^4^ cells/mL were incubated with and/or without the tested compounds in 6-well plates in a final volume of medium—2 mL. Both collagen and protein concentration in cells and medium was analyzed at concentrations of 10 µM and 1 µM for K and 1 µM and 0.1 µM for BA. Cells were lysed by freezing and thawing twice at −80 °C.

### 2.3. Tested Phytohormones

K and BA were stored in a freezer and added to the culture medium in amounts that gave final concentrations of 100 µM, 10 µM, 1 µM, and 0.1 µM. Control cells were incubated without the tested phytohormones.

### 2.4. Cytotoxicity

In order to analyze the potentially cytotoxic effect of the studied compounds—K and BA—a luminescent assay, CellTiter-GloTM 2.0 Assay (Promega, Madison, WI, USA), was used. Control, untreated cells, were incubated without tested compounds in DMEM only. The analysis was performed according to the manufacturer’s protocol. Blank samples only contained cell culture medium, without cells. GloMax-Multi Microplate Multimode Reader was used to measure luminescence. The study was performed in triplicate taken to ensure consistent results were obtained.

### 2.5. Wound-Healing Assay

This method is used to assess the activity of substances influencing cell motility and proliferation in vitro [10]; it is used in studies on wound healing and angiogenesis. In this way, how stimulation with specific cytokinins affects cell motility was studied. Cultures at the level of 1 to 4 passages were used for the experiment. Cells were cultured in culture plates until a uniform layer of cells was obtained (full confluence). Solutions of medium plus FBS and cytokinin solutions with specified concentrations were prepared. All solutions should be filtered to maintain sterility. A line was drawn with a marker on the bottom of the culture vessels. Using sterile automatic pipette tips, a scratch was made in the uniform layer of cells, and the detached cells were removed by washing the plates twice with PBS solution. Medium and phytohormones were added to the cultures prepared in this way at the concentration considered the most optimal. Then, photos were taken after 6, 12, and 24 h. After each photo and measurement of the decreasing scratch, the medium with the addition of phytohormones was changed.

### 2.6. Determination of Total Protein Content in Cells

Preparation of samples for total protein determination: after removing the medium, the cells were washed with PBS. Then, the cells were scraped off and centrifuged for 10 min at 1000 rpm. After decanting the supernatant, the cells were washed with 1 mL of 1 M NaOH and transferred to a boiling water bath for 10 min. Then, 1 mL of distilled water was added and the diluted extract was used for further analysis. Blank samples contained 1 M NaOH solution. The Lowry method was used for the analysis [11].

### 2.7. Determination of Collagen Content in Culture Medium and Cells

This method is based on spectrophotometric observation of the binding of Sirius red dye in saturated picric acid to fibrillar collagen (type I to V), especially to its Gly-X-Y fragment of the helical structure [12]. The method is used to measure collagen content in the culture medium and in cells.

Standard collagen solutions were prepared by dissolving collagen in 0.5 M of acetic acid to obtain concentrations of 0 μg, 5 μg, 10 μg, 20 μg, 30 μg, and 50 μg. Then, 1 mL of 0.1% dye solution was added to standard solutions, blank (0.5 M acetic acid solution) and test samples of 50–100 μL and mixed gently at room temperature for 30 min. Samples were centrifuged at 10,000 rpm for 5 min to pellet collagen. Supernatant was carefully removed so as not to disturb the pellet. Next, 1 mL of 0.1 M HCl was added to each tube to remove unbound dye. Samples were centrifuged again at 10,000 rpm for 5 min. Then, 1 mL of 0.5 M NaOH was added to each tube and vortexed to release bound dye. Absorbance was read at 540 nm. Collagen content in the test samples was read using a standard curve introduced into the spectrophotometer.

### 2.8. Statistical Analysis

The results are presented graphically as a boxplot and as column plots showing the mean ± standard deviation (n = 3). ANOVA analysis was used to compare the means obtained for live and dead cells, and significant differences were evaluated by Dunnett’s test, where (*), (**), and (***) are at *p* < 0.05, *p* < 0.01 and *p* < 0.001, respectively. Relationships between the studied parameters are presented as a biplot. MS Excel, Statistica 13.3, and R package 4.4.1 (ggplot2) were used for calculations and data visualization. Data regarding protein content and collagen content in cells were normalized by measuring total cell number to exclude the risk that variable cell numbers were lysed.

## 3. Results

### 3.1. BA and K Cytotoxicity

**Figure 1:**
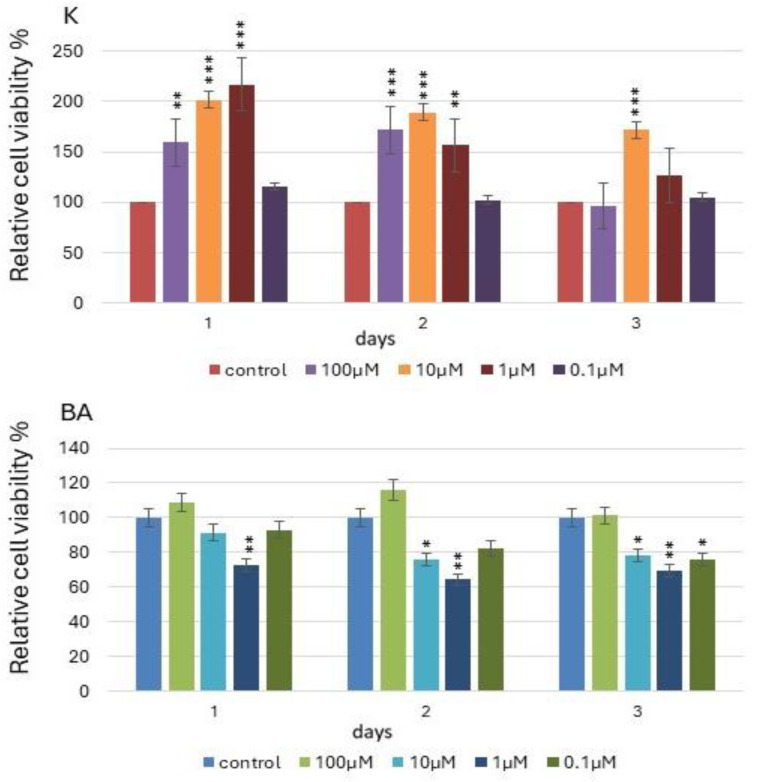
Relative viability of cells as a result of 3-day incubation with K and BA in selected concentrations measured by using CellTiter-GloTM. *, ** and *** indicate significant differences assessed by Dunnett’s test between cells treated with K and BA at different concentrations and control.

Relative cell viability shows a certain noticeable tendency to change depending on both time and the concentration of each of the tested compounds (Figure 1). In the case of K, these changes are statistically significant, especially in the first three days of the experiment, when we observe the highest increases in cell viability. K concentrations of 10 μM and 1 μM proved to be particularly stimulating for this parameter. In the following days, the increases are not as high, and at the highest tested concentration of 100 μM, decreases in the tested parameter were noted. In the case of BA, however, small increases in the level of cell viability are not statistically significant. In the case of BA, a tendency is also noticeable indicating intensive stimulation by higher concentrations (100 μM), while the opposite situation occurred for K, where the lower concentration (10 μM) proved to be more stimulating. It is noticeable in both cases that the last days of the experiment for both tested compounds show a downward trend. This may be related to the high cell density at the final stage of the procedure.

### 3.2. Total Protein Content in Cells

**Figure 2:**
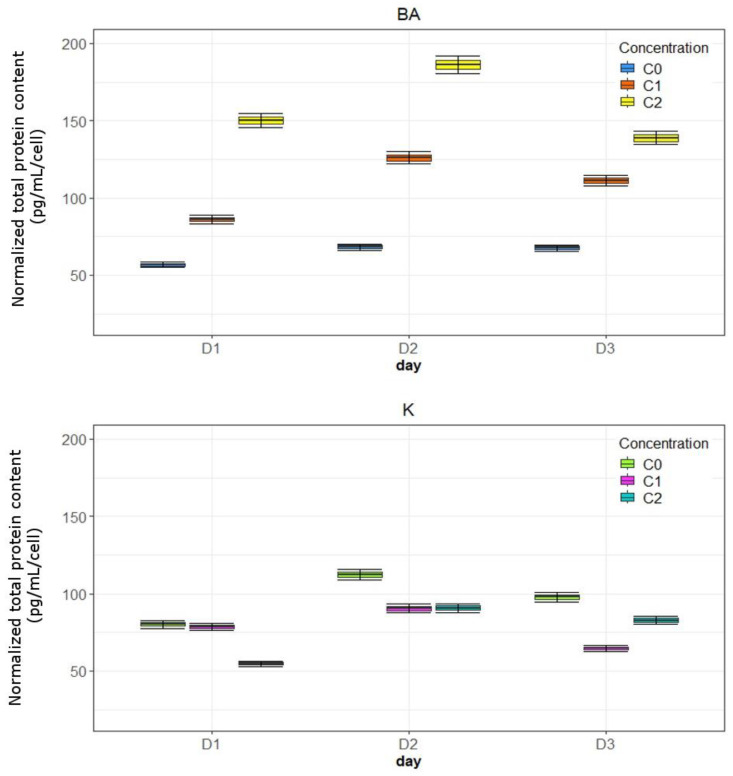
The influence of K and BA on total protein content in fibroblasts during 3-day incubation. BA: C0—control; C1—1 µM; C2—0.1 µM; K: C0—control; C1—10 µM; C2—1 µM.

Kinetin caused an increase in protein content, which was observed especially under the influence of the higher analyzed concentration on each day of the experiment (Figure 2). BA had a stronger effect on the tested parameter at the lower tested concentration (Figure 2). The highest increase in the studied parameter had already been observed on the first day of the experiment for the concentration of 1 µM BA by about 80% compared to the control and for the same concentration by about 70% comparing to untreated cells after 48 h of incubation. In the case of none of the cytokinins, a decrease in studied parameter level below the control level was observed. The analyzed substances had a stimulating effect on this parameter, but the obtained results are not statistically significant.

### 3.3. The Amount of Collagen in DMEM and in Fibroblasts

**Figure 3:**
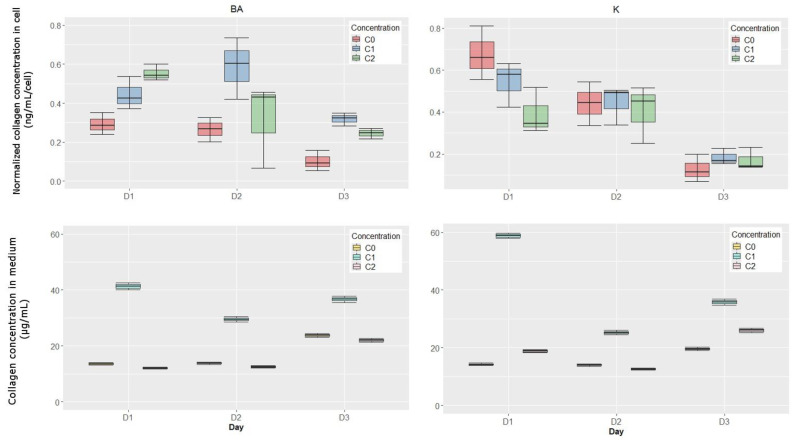
The influence of K and BA on collagen content in cells and in the culture medium during 3-day incubation. BA: C0—control; C1—1 µM; C2—0.1 µM; K: C0—control; C1—10 µM; C2—1 µM.

Kinetin stimulates collagen biosynthesis and its secretion into the culture medium to a greater extent. At a concentration of 10 μM after 24 h of incubation, a significant increase in collagen content was observed, by about 320% compared to untreated cells. (Figure 3). In the case of BA, significant stimulation of the increase in the presented parameter level in DMEM was noticed at 1 μM. (Figure 3). The presented results regarding collagen concentration in the culture medium with the addition of the studied cytokinins are statistically significant.

In the culture treated with K, the highest collagen content in cells was noticed after 72 h of incubation. At a concentration of 10 μM it was 157%, and at 1 μM—66.34% compared to the control. BA caused a significant increase in the studied parameter level in cells on the third day. The results showing changes in the collagen content in cells are not statistically significant.

### 3.4. The Effect of Cytokinins on Fibroblast Migration and Proliferation

**Figure 4:**
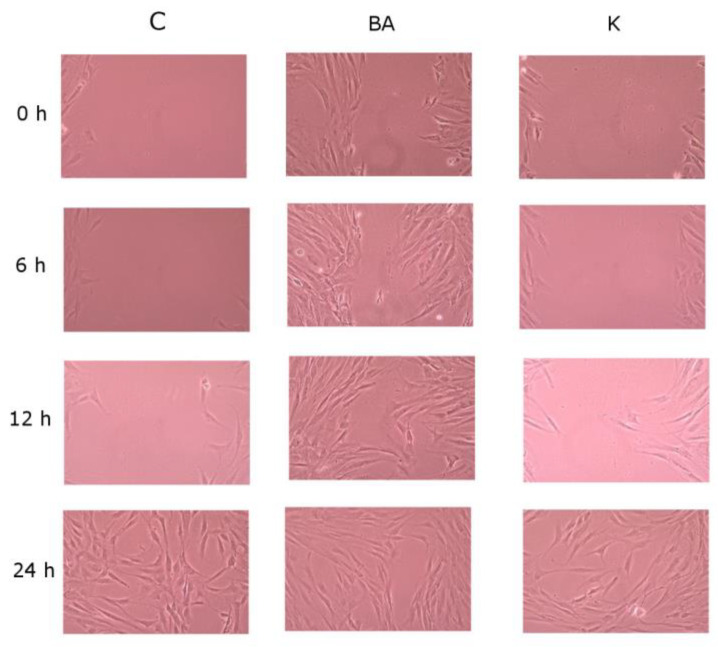
Effect of K (1 µM) and BA (0.1 µM) on fibroblast migration analyzed in the wound-healing assay.

Regarding 1 μM of K, it stimulated cell migration and the most intense stimulation of proliferation was observed. The slowest cells colonized the “wounded” site when stimulated with BA at a concentration of 0.1 μM (Figure 4). In this study, no proliferation inhibitors were used, therefore the obtained results also indicate increased cell proliferation under the influence of the tested compounds.

## 4. Discussion

Changes in hormone production in the body, especially in women, are frequent and not only correlate with aging, but also with different pathological conditions. This usually has a very significant impact not only on the functioning of the skin, but also on the functioning of the entire body. The data from the literature indicated that the external use of hormones in prescription composition is not limited to the place of application of the preparation and can cause additional effects in relation to the entire body [13,14]. Therefore, in many countries, including Poland, it is prohibited to use estrogen hormones of animal origin in over-the-counter preparations. Phytohormones are substances, which are characterized by biological activity that have the nature of chemical signals, produced by specific tissues and transported to areas where they stimulate growth and others metabolic pathways. The structural and functional similarity of phytohormones and human hormones generates the possibility of their use in the medical field. However, the fundamental difference is the very wide spectrum of action that characterizes phytohormones. They can affect various metabolic processes, and the factors determining the type and action of a given hormone depend in particular on the concentration, location, and interactions with other phytohormones [15,16,17]. Phytohormones, in addition to their effect on receptors specific to human hormones, are characterized by very strong antioxidant properties. They are considered as free radical scavengers and compounds which may prevent or even stop cell mutagenesis, thereby exhibiting anticancer effects. All of the extremely beneficial above-mentioned aspects of the effect of phytohormones on the human body indicate that there is a significant need to investigate this further. Such a group of compounds includes cytokinins such as K and BA.

**Figure 5:**
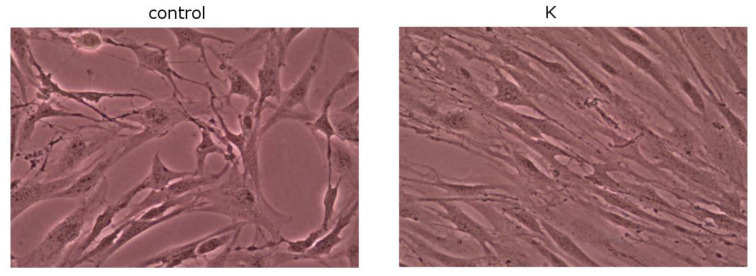
Effect of K (1 µM) on fibroblasts density and morphology.

Kinetin-stimulated cells do not undergo the morphological changes connected not only with aging but also with different pathological conditions as rapidly as untreated cells (Figure 5). Stimulated cells are characterized by higher density, indicating more intensive proliferation, and regular shape compared to the control. K-treated cells were spindle shaped, oriented regularly and had typical fibroblast morphology. The shape of the nuclei was rather regular and ovoid. When cells became confluent, they were characterized by a typical appearance. Control culture cells were characterized by different, irregular shapes with cytoplasmic projections.

Healthy human skin fibroblasts isolated from healthy adult donors cultured in vitro exhibit an elongated and spindle-shaped shape. At the stage of confluent culture, they are characterized by a specific appearance consisting of being quite thin and elongated and parallel to each other. With subsequent passages, the cells become heterogeneous in appearance, large, flattened, amorphous, and filled with residual lysosomal bodies and contain several cell nuclei. It was noticed, which is consistent with reports from the literature [18,19], that cells stimulated with kinetin do not undergo above mentioned morphological changes to the same extent as untreated cells. Our own studies have shown that even on fifth day, the cells maintain spindle shape, arrange themselves parallel to each other and create a single layer. Kinetin also affects the speed of fibroblast migration, which was presented as a result of the wound-healing assay. A scratched “wound” created in a confluent culture allows the cells to inhabit the place that is to represent the torn tissue. A 1 μM K treatment means that after 24 h the “wound” is almost completely covered with cells.

Our own studies have shown that kinetin has a more stimulating effect than benzyladenine on the cells’ proliferation and protein content, especially after 24 h of incubation. The literature reports also indicate an increase in protein content in fibroblast cultures stimulated with kinetin [18,20]. The effect of kinetin on the collagen concentration has not been studied so far and there is no literature data on this subject. Our own studies indicate that this hormone in particular stimulates collagen synthesis, which is clearly noticeable at a concentration of 10 μM, as its content increases both in the culture medium and in the cells. A significant increase in collagen content in DMEM was noted after 24 h and 48 h of incubation with the tested compound. On the third day, an increase in the amount of this protein in the cells was noted, followed by a decrease even below the control value on the fourth day, but at the same time an increase in the amount of collagen in the culture medium occurred. This may suggest that after synthesis, this protein was secreted into the culture medium. On the last day, when a decline in the collagen concentration was observed in the cells, its amount in the medium increased. Since the literature data [20,21] indicate that kinetin can act at the transcriptional, translational, post-translational and metabolic levels, it is not clear at what point in the cell cycle collagen synthesis can be induced. The results we obtained confirm to some extent the beneficial effect of kinetin on the basic components of the extracellular matrix. However, it should be remembered that these are the results of in vitro studies, which, in our opinion, only indicate the direction in which these studies should be continued. According to Kimura T. et al., the skin treated with kinetin showed significantly better morphological organization of its basic cellular components than the control [22]. Before kinetin treatment, collagen fiber thickenings with an irregular arrangement were visible in the dermis. After 50 days of kinetin exposure, collagen fibers regained their proper size and density of weave and appropriate distribution of collagen in skin. An improvement in the extracellular matrix components function and structure was noticed throughout the time of kinetin exposition. The presented data were obtained from the experiments with Mexican hairless dogs [22]. Our results, in this context, are both confirmation and an indication of the direction of future research regarding studies of kinetin activity in skin.

Our own studies confirmed that benzyladenine did not stimulate cell proliferation. At concentrations of 1 μM and 0.1 μM, it had the weakest effect on inhibiting divisions in fibroblasts, which is why these concentrations were used for further studies and to check the effect of benzyladenine on other parameters such as total protein and collagen content [8]. It is also worth noting that BA shows greater cytotoxicity at lower concentrations. The obtained results may be similar to those obtained by Ishii Y et al. and Mlejnek P [23,24]. They showed induction of cell differentiation in the concentration range of 25–100 µM, but lethal effect on human cells at much lower concentrations in which riboside forms of cytokinin induced apoptosis, i.e., cell death. In fibroblasts, benzyladenine acts through analogical mechanisms of inhibition cells divisions as in cancer cells, i.e., it is a strong and specific inhibitor of kinases CDK. They are important factors in the mitosis phase and in the early phases of cell division (interphase) [25,26]. Although benzyladenine did not stimulate significantly cells proliferation and few fibroblasts in mitosis were observed in culture, morphologically, cells stimulated with this hormone maintain their elongated shape characteristic of cells in the early phase of growth. This is consistent with the data of Johnson G.S. et al. that N-6 adenine derivatives affect cell shape by elongating them [27]. In addition, compounds including N-6-benzyladenine cause a significant slowdown in cell migration and increase their adhesion to the substrate. This is in accordance with our data, which indicate that benzyladenine at a concentration of 0.1 μM stimulated cell migration the weakest in the WOUND ASSAY test. Similar effects on cell shape, migration and adhesion were observed during the incubation of cells with potential phosphodiesterase inhibitors, e.g., 1-methyl-3-isobutylxanthine, which caused an increase in the intracellular cAMP level. Available data indicate that benzyladenine and its derivatives act at the cellular level as selective inhibitors of phosphodiesterases (PDEs), which are enzymes that mediate cellular signaling through the hydrolysis of cAMP and cGMP [28,29]. Since PDEs are key enzymes involved in inflammatory processes, the PDE-inhibiting activity of BA seems to be interesting in the context of diseases associated with inflammation. This action is also related to the effect of BA on TNF-α synthesis, because available data indicate that PDE inhibitors are also anti-TNF-α agents used, among others, in the treatment of asthma, atopic dermatitis, chronic obstructive pulmonary disease, and rheumatoid arthritis [30,31,32].

However, the available literature indicates that benzyladenine stimulates protein kinase A, but via a cAMP-independent mechanism [33,34]. It can be stated that this is to some extent consistent with our results, which indicate a stimulating effect of benzyladenine on collagen synthesis. No decreases in protein content below the control level were observed. The examined content of collagen secreted by cells into the culture medium indicates a strong stimulating effect of benzyladenine at a concentration of 1 μM, especially after 24 h of incubation. After 72 h of incubation, there is a decline, however, the level is still above the control. At the same time of incubation, a significant increase in the collagen content in the cells was noted, while on days 1 and 4 the amount in the cells was significantly lower than on day 3. Based on the available data on BA, it can be assumed that BA activates protein kinase A and stimulates the synthesis and secretion of collagen into the culture medium already in the first two days of culture, after which the content of this protein in the cells increases (on the third day), and then the synthesized collagen is secreted into the medium on the fourth and fifth day. At that time, we observe a significant increase in its content in the culture medium. In fibroblasts under the influence of benzyladenine at a concentration of 0.1 μM, an increase in the collagen content was observed on the first, third and fifth day, and on the second and fourth day, slight decreases in the content of the tested protein. The presented data may to some extent confirm the hypothesis of Swaney J.S. et al. that high levels of cAMP inhibit collagen synthesis [35]. It can be speculated that the induction of collagen synthesis by BA occurs via a metabolic pathway independent of cAMP, but only via the activation of protein kinase A, which in turn occurs via interaction with TGF-β, which stimulates collagen synthesis in cells [36,37].

**Figure 6:**
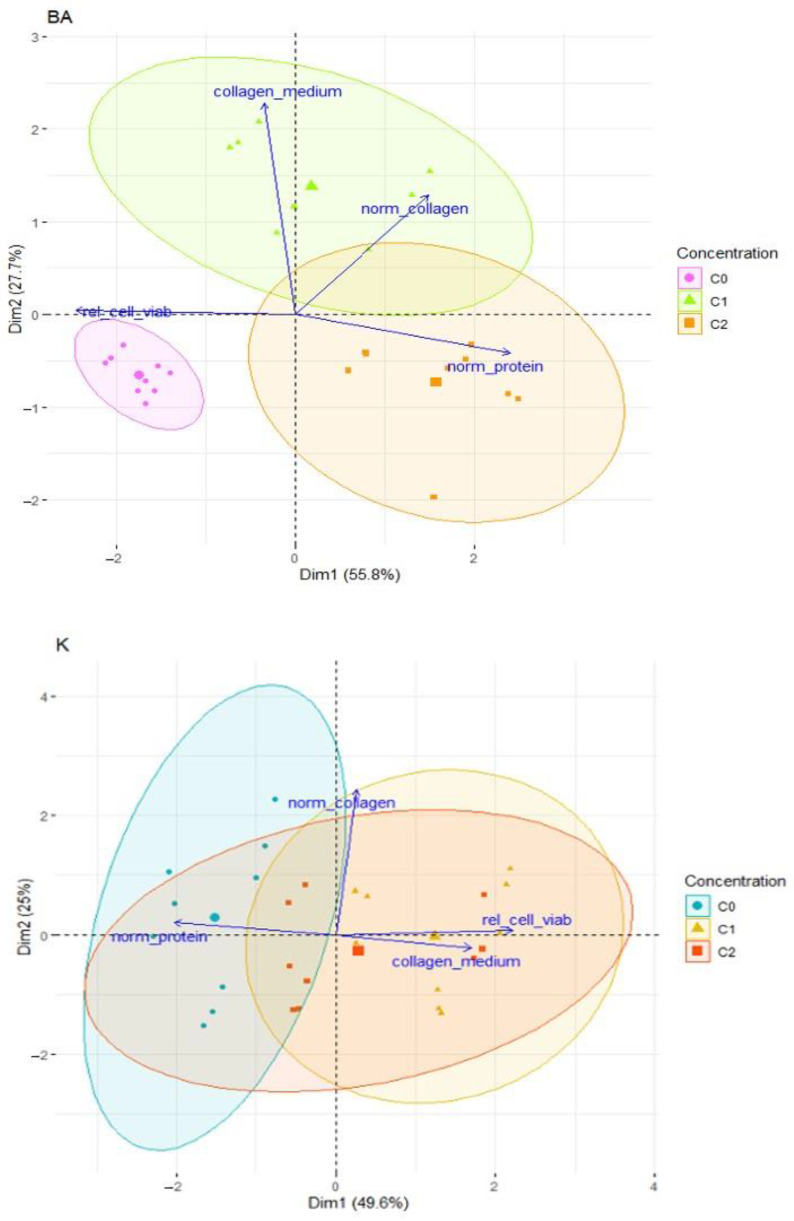
The relationships between the studied parameters after the application of BA and K at selected concentrations, presented as a biplot.

The tested plant hormones exhibit a positive effect on the proliferation and action of the fibroblasts. They induce the biosynthesis of total protein, collagen, and have a positive effect on the oxidative stress level and the mobility of fibroblasts [8]. In the case of kinetin, a positive correlation was observed between the parameters tested, in particular the number of living cells, the content of total protein and the content of collagen (Figure 6). This indicates an obvious positive effect of kinetin on the functioning of skin cells and their synthesis of basic building proteins of the skin and, consequently, on the reduction in visible symptoms of skin aging. In the case of benzyladenine, a slightly different correlation was observed (Figure 6). The decrease in the number of living cells significantly affected the results obtained for collagen and total protein. Hence, there was no such significant correlation of the parameters studied as in the case of kinetin. Collagen and elastin fibers under the influence of kinetin regain their proper structure, and excessive skin pigmentation is also reduced. Kinetin is effective in low doses, as confirmed by our own studies on fibroblasts, but its effectiveness depends to a large extent on the application time. It has been observed that this plant hormone does not exhibit allergic potential and it can be applied on sensitive skin [38,39,40]. The effect of kinetin, especially its riboside forms, on cancer cells is also known and confirmed, where it is a stimulator of apoptosis. Kinetin nucleosides and ribonucleosides have a stimulating influence on cancer cell apoptosis, while not damaging healthy cells [41,42,43]. It seems, taking into account all the above-mentioned positive effects of the application of the tested phytohormones in vitro, that after the implementation of in vivo studies, it will be possible to plan clinical applications of the tested compounds. They seem to be promising not only in terms of skin aging, but most of all in terms of diseases associated with changes in the connective tissue and with respect to scarring. Thinking about the development of research on cytokinins, the direction of treating inflammatory conditions associated with tissue loss, necrotic conditions in the connective tissue, and wounds seems interesting.

## 5. Conclusions

In this study, we describe for the first time the effect of two selected cytokinin compounds on fibroblast activity. Furthermore, we have shown a stimulating effect of benzyladenine and kinetin on the production of collagen, which is the basic structural protein of the skin, in fibroblasts. Based on our data using a single human cell line, we conclude that it is necessary to investigate the metabolism of BA and K using many different cell lines from different tissues to expand our knowledge of the endogenous metabolism and action of cytokinins in human tissue systems. At present, our findings are limited to a single cell line and selected assays; therefore, additional studies conducted in different human cell lines are important to lay the foundation for elucidating the metabolism of the studied compounds in the human organism. Moreover, the examined and presented collagen content changing under the influence of cytokinins and the proliferative and migratory abilities of fibroblasts observed in this study need to be verified in future studies to confirm whether cytokinins can really be considered as substances supporting the functioning of human skin under physiological and pathological conditions.

It can be concluded that kinetin is a promising agent that still awaits further toxicological and efficacy tests. On the other hand, benzyladenine, whose derivatives have proven anti-carcinogenic effects, can be used as a therapeutic substance in inflammatory conditions as well as in skin diseases associated with pigmentation disorders. There is a certain correlation between carcinogenesis in humans and in plants. Factors that play an important role in the regulation of differentiation and development in plants can also affect the differentiation of human cells, both healthy and pathologically changed, through the signal transduction system and therefore can be clinically useful in the treatment of various disease states. It seems that they may prove useful in pathological conditions associated with both oxidative stress and quantitative and qualitative changes in the structural proteins of connective tissue. The effectiveness of their action presented in this work in fibroblasts as a skin cell in vitro model may indicate their therapeutic potential; however, in addition to molecular in vitro studies, a direct assessment of cytokinins’ toxicity in vivo in an animal model should be conducted.

## Data Availability

The data presented in this study are available on a reasonable request from the corresponding author.
